# Self-induced Widespread emphysema in the first psychosis episode in a migrant worker: case report

**DOI:** 10.1192/j.eurpsy.2025.1459

**Published:** 2025-08-26

**Authors:** A.-T. Rimmel, R. Nefzi

**Affiliations:** 1 PSYCHIATRIE, RFSM, BULLE, Switzerland

## Abstract

**Introduction:**

In psychosis, self-mutilation reveals an intense alteration in body perception. This is even truer during the first psychotic episodes when one is overcome by delirium and hallucination. It often leads to physical disability particularly in ocular, genital, and limb regions.

Migrant have shown to present a higher risk of psychosis and schizophrenia. With the language barrier burden, self-mutilation may appear, as an attempt to remove from the body what cannot be verbally expressed.

**Objectives:**

To describe the clinical presentation of an acute psychotic decompensation associated with unusual somatic symptoms (generalized emphysema), highlighting the importance of integrated psychiatric and somatic care.

To emphasize partial adherence to delusional ideas to distinguish intrapsychic thoughts from auditory hallucinations and refine the diagnosis.

**Methods:**

Presented the case of a 25-year-old Eritrean male, living alone in Switzerland for 8 years who self-induced a widespread emphysema ( Pneumomediastinum, orbital left pre- and post-septal, intracanal extradural, pre- and retroperitoneal extending to the pelvis) by attempting to unblock his nose with his right little finger secondary to a Command hallucination in a first psychosis episode.

**Results:**

The patient has not given news to his friends for 3 weeks. When visiting him they noticed that his apartment was unkempt and messy. The patient had a strange mystical speech and explained hearing a voice that tries to command him. He denied suicidal intent.

Emergency exam revealed Subcutaneous emphysema and the A computed tomography body scan displayed a generalized soft tissue emphysema extending from the left temporal region, left pre- and post-septal orbital emphysema, throughout the neck spaces, intracanal extradural (cervical spine), dissecting

The mediastinum with scissural extension, probable emphysema component outside the parietal pleura (pneumothorax component is not excluded) extending preperitoneally and retroperitoneally to the level of the pelvic floor and extends along the iliac axes and into the left testicle. Also present, soft tissue emphysema in the left upper limb, up to the wrist. No pharyngolaryngeal, tracheal or esophageal perforation visible on CT. Otorhinolaryngology origin too small to be visualized.

The patient was hemodynamically *stable,* the laboratories were normal and no clinical evidence was found to consider an organic mental disorder. No drug history was found.

The mental status revealed mystical delusion with intrapsychic hallucination ordering him to evacuate the feeling of chest tightness he had felt with unblocking his nose. The diagnosis of schizophrenia spectrum disorder was retained.

**Image:**

**Figure:**
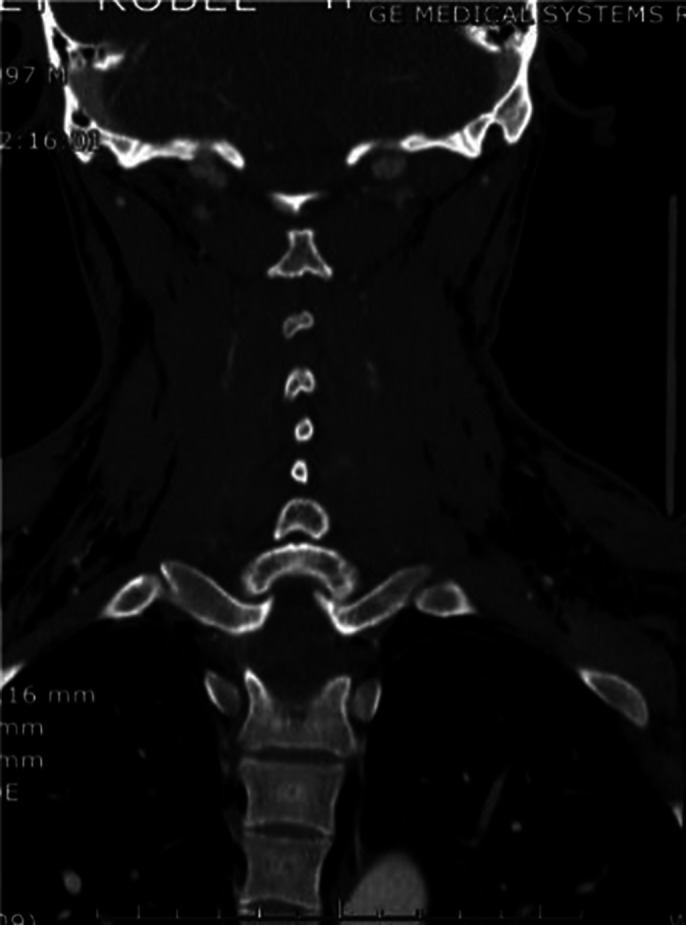

**Image 2:**

**Figure:**
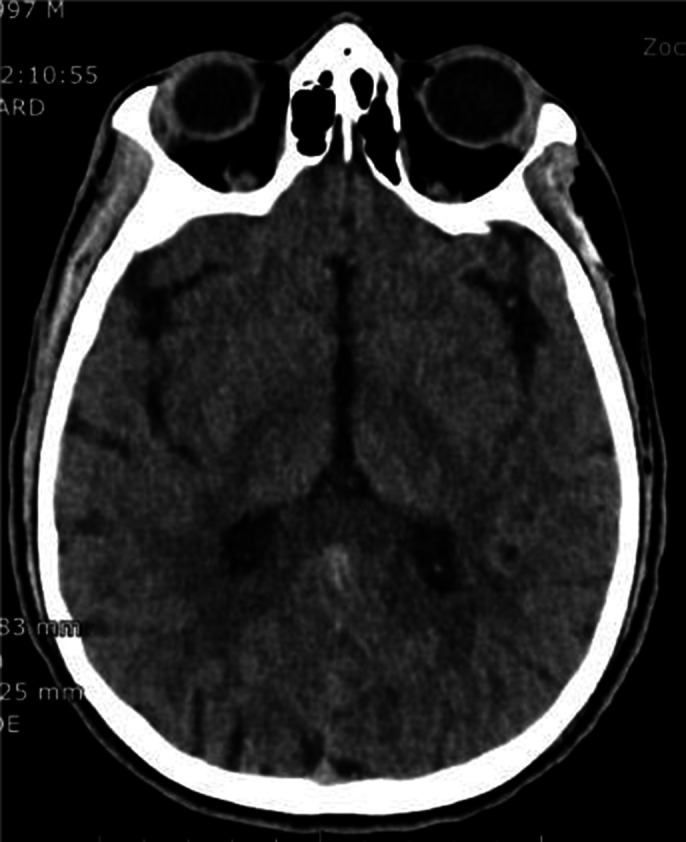

**Image 3:**

**Figure:**
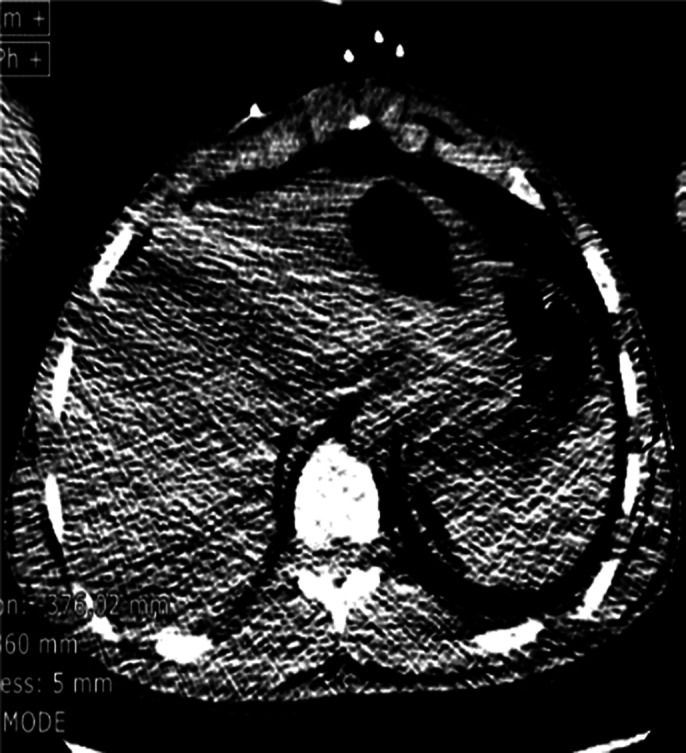

**Conclusions:**

The case confirmed the importance of recognizing physical symptoms in a psychiatric context to avoid severe complications and the impact of multidisciplinary assessment.

**Disclosure of Interest:**

None Declared

